# Localization and Dynamics of the Methionine Sulfoxide Reductases MsrB1 and MsrB2 in Beech Seeds

**DOI:** 10.3390/ijms22010402

**Published:** 2021-01-02

**Authors:** Natalia Wojciechowska, Agnieszka Bagniewska-Zadworna, Julia Minicka, Kornel M. Michalak, Ewa M. Kalemba

**Affiliations:** 1 Institute of Dendrology, Polish Academy of Sciences, Parkowa 5, 62-035 Kórnik, Poland; 2 Department of General Botany, Institute of Experimental Biology, Faculty of Biology, Adam Mickiewicz University, Uniwersytetu Poznańskiego 6, 61-614 Poznań, Poland; agabag@amu.edu.pl (A.B.-Z.); kornel.m.michalak@amu.edu.pl (K.M.M.); 3 Department of Virology and Bacteriology, Institute of Plant Protection, Władysława Węgorka 20, 60-318 Poznań, Poland; j.minicka@iorpib.poznan.pl

**Keywords:** *Fagus sylvatica*, storage, aging, methionine reduction, seed viability, immunocytochemistry

## Abstract

Beech seeds are produced irregularly, and there is a need for long-term storage of these seeds for forest management practices. Accumulated reactive oxygen species broadly oxidize molecules, including amino acids, such as methionine, thereby contributing to decreased seed viability. Methionine oxidation can be reversed by the activity of methionine sulfoxide reductases (Msrs), which are enzymes involved in the regulation of many developmental processes and stress responses. Two types of Msrs, MsrB1 and MsrB2, were investigated in beech seeds to determine their abundance and localization. MsrB1 and MsrB2 were detected in the cortical cells and the outer area of the vascular cylinder of the embryonic axes as well as in the epidermis and parenchyma cells of cotyledons. The abundances of MsrB1 and MsrB2 decreased during long-term storage. Ultrastructural analyses have demonstrated the accumulation of these proteins in protein storage vacuoles and in the cytoplasm, especially in close proximity to the cell membrane. In silico predictions of possible Msr interactions supported our findings. In this study, we investigate the contribution of MsrB1 and MsrB2 locations in the regulation of seed viability and suggest that MsrB2 is linked with the longevity of beech seeds via association with proper utilization of storage material.

## 1. Introduction

Beech (*Fagus sylvatica* L.) is one of the primary species observed in forest ecosystems in Europe, the total area of which is estimated at 14–15 Mha [1,2]. This species is propagated primarily by seeds, which are classified to the intermediate category because they exhibit loss of viability during long-term storage [3]. Storage of beech seeds is required because beech seed production is very irregular, and a good harvest year occurs once in 5–10 years [4,5]. Therefore, proper seed storage conditions that ensure high viability are crucial for plant nurseries and the forest industry. Optimal storage conditions established for beech seeds include a water content (WC) range of 7.8 to 11.5% and a temperature range from −10 °C to −20 °C [6].

Reactive oxygen species (ROS) are crucial factors that influence the loss of viability in seeds [7]. Increased ROS concentration in plant tissues results in cellular damage involving decreasing activity of the antioxidant system; lipid peroxidation; and oxidative damage to DNA, RNA, and protein, and it finally leads to cell death [8,9]. ROS accumulation has been determined to be an important factor that affects beech germination capacity after long-term storage [10]. Interestingly, protein oxidation via carbonylation has been determined to contribute to the lowered viability of naturally aged beech seeds [11].

Oxidation affects the integrity of amino acids, causes changes in protein structure, and eventually affects protein activity [12]. Methionine (Met) is one of the major amino acids susceptible to oxidation, which causes it to convert to methionine sulfoxide (MetO) [13]. However, MetO can be easily reduced back to Met through the activity of methionine sulfoxide reductase (Msr) enzymes. There are two types of Msrs, MsrA and MsrB, which are specific to the S- and R-diastereomers of MetO, respectively [14]. The A and B types of Msrs exhibit no sequence or structure similarity [15,16,17]. The majority of Msr enzymes, including MsrB2, possess two redox-active cysteines [18,19]. However, some MsrB proteins, such as MsrB1, lack the second resolving cysteine and employ other mechanisms for their regeneration [20,21].

The presence of Msr and their roles in protection against oxidative stress have been confirmed in most organisms, including bacteria, yeast, mammals, and plants [20]. In the 1960s, Msr enzymatic activity was first reported in plants [22], and in subsequent decades, many studies have confirmed the involvement of Msrs in the regulation of various plant processes. Plants are characterized by an extraordinarily large number of Msr isoforms; thus, their role may be more complex than in other organisms [12,19]. The majority of research has focused on investigating the role of Msr in the stress response and for both abiotic [23,24], and biotic [25,26] stresses. Moreover, an increasing number of studies have suggested that Msrs may participate in developmental processes, including seed maturation [27], seed desiccation [28], seed longevity [29], and senescence and maturation of fruits [30,31].

Msr proteins are detectable in almost all plant organs; however, the expression of specific Msr isoforms in a given plant organ strictly depends on the function of the organ [13]. The occurrence of Msrs has been confirmed in roots, leaves, flowers, flower buds, pollen, stems, seeds, and seedlings (as reviewed in Rouhier et al. [12]). Young leaves, photosynthetic organs, and floral buds have been identified as specific organs for both MsrB1 and MsrB2. Interestingly, microarray analyses revealed that the *MsrB2* gene was also expressed in seeds [32]. Proteins possessing signal peptides in their amino acid sequence are transported to their appropriate destinations in cells via protein targeting [33]. The generation of different mRNA forms, posttranslational modifications, and signal exposing/masking switching mechanisms enable multiple protein localizations [34]. MsrB1 and MsrB2 are believed to be characteristic proteins of photosynthetic tissues, and their localization in chloroplasts is primarily related to the abundance of ROSs that are generated by those organs [20,23]. Importantly, MsrB1 and/or MsrB2 have been reported not to be present in tissues without chloroplasts [20,29,35].

In this study, we hypothesize that MsrB in mature beech seeds is involved in successful protection against oxidation in seeds requiring long-term storage and accurate damage repair when subjected to germination. Thus, to determine the role of MsrBs in preserving seed longevity, we investigated the presence of MsrB1 and MsrB2, assumed to be plastidial proteins, in stored beech seeds despite the absence of active chloroplasts. We employed detection and localization techniques to confirm the presence of the tested proteins in both cotyledons and embryonic axes, and together with ultrastructure analyses, these experiments provide new data on the cellular distribution of MsrB proteins.

## 2. Results

### 2.1. Immunodetection of MsrB1 and MsrB2

**Figure 1:**
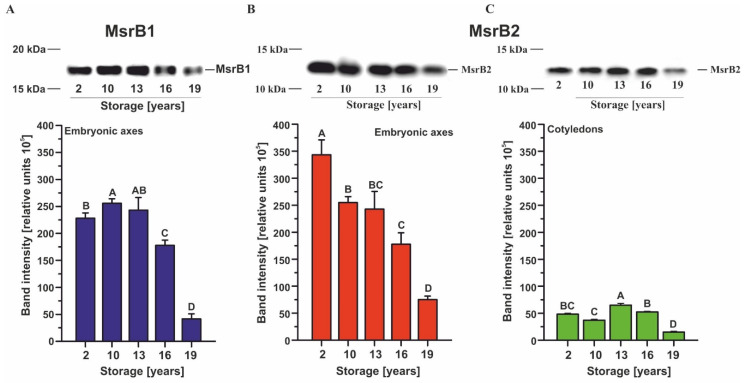
Immunoblot analyses and densitometric analysis of MsrB1 (**A**) and MsrB2 (**B**,**C**) proteins in the embryonic axes and cotyledons of dry beech seeds stored for 2–19 years: the data represent the means of three independent replicates ± the standard deviations. The same letters indicate groups that are not significantly different according to Tukey’s test.

MsrB1, assumed to be a 17-kDa protein, was detected in embryonic axes, and its abundance decreased during long-term storage, which was particularly noticeable after 16–19 years of storage (Figure 1A). MsrB1 could not be detected in beech cotyledons through Western blot analysis. In contrast, MsrB2 was detectable in both embryonic axes (Figure 1B) and cotyledons of beech seeds (Figure 1C). Particularly in embryonic axes, MsrB2 displayed an almost linear decrease in abundance during storage (R = −0.9361, *p* < 0.0001), whereas cotyledons exhibited a significantly decreased amount of MsrB2 in seeds stored only for the longest period of time.

### 2.2. Tissue Localization of MsrB1 and MsrB2 in Beech Seeds

**Figure 2:**
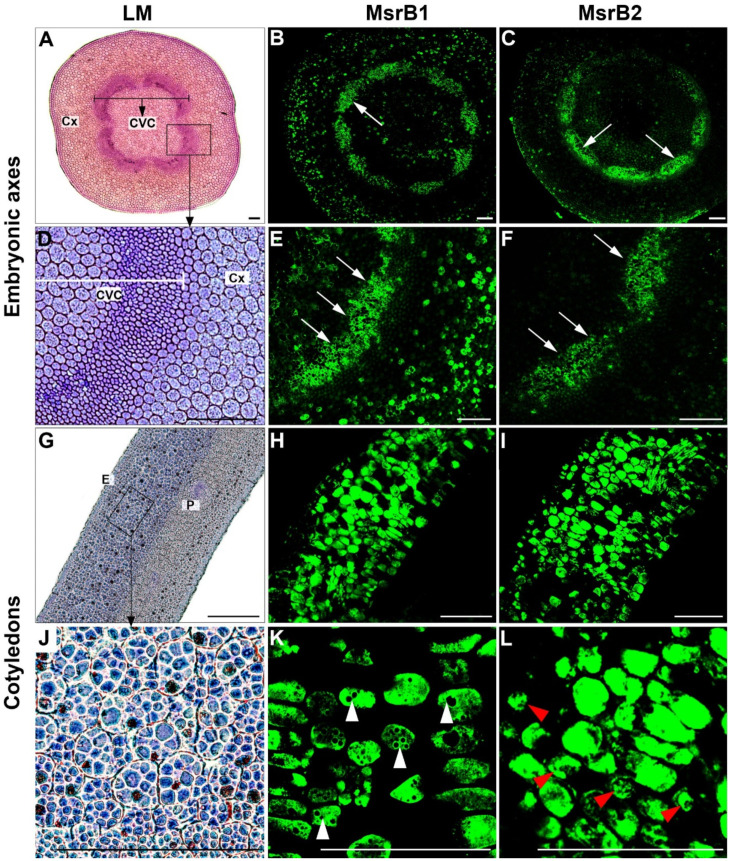
Structure and localization of MsrB1 and MsrB2 in embryonic axes (**A**–**F**) and cotyledons (**G**–**L**) of beech seeds: Cx—cortex precursor cells, CVC—central vascular cylinder cells, P—parenchyma cells, E—epidermis, and LM—light microscopy; scale bars: 100 µm. Arrows indicate the localization of MsrB1 and MsrB2 in outer areas of CVC, white arrowheads indicate the localization of MsrB1 around the spherical structures, red arrowheads indicate the localization of MsrB2 in irregular spots.

The immunolocalization reaction was combined with the tyramide signal amplification (TSA) technique to increase the intensity of the acquired signal and to detect even small amounts of proteins. Moreover, histological analyses were also performed to characterize the anatomical structure of the examined organs and to determine the locations of proteins in specific tissues and regions of the embryo (Figure 2A,D,G,J). An intensive signal of MsrB1 was detected in the outer areas of central vascular cylinder cells corresponding to developing conducting tissues of the beech embryonic axis (Figure 2B,E, arrows). Anatomical studies demonstrated that the signal detected in conductive tissue areas corresponded to cells with features and arrangement characteristic of xylem (Figure 2A,D). In addition, the signal was also present in the few cortex precursor cells and in cells located in the middle of the central vascular cylinder in embryonic axes (Figure 2B,E). In cotyledons, MsrB1 was localized in the epidermal layer as well as in the majority of parenchyma cells, in which it often filled the entire cell (Figure 2H,K) or surrounded the spherical structures (Figure 2K, white arrowheads).

The analyses of MsrB2 protein localization in the embryonic axes demonstrated a signal distribution pattern similar to that observed for the MsrB1 protein, but the fluorescence intensity was slightly lower. The highest signal was detected in the outer areas of the central vascular cylinder (Figure 2C,F, arrows). Furthermore, the MsrB2 protein was located in several cortex precursor cells (Figure 2C). In cotyledons, the signal was observed in most cells of the epidermis and parenchyma. The signal distribution was different for individual parenchyma cells for which fluorescence was detected in entire cells (Figure 2I,L) or in irregular spots (Figure 2L, red arrowheads).

### 2.3. Subcellular Localization of MsrB1 and MsrB2 in Beech Seeds

**Figure 3:**
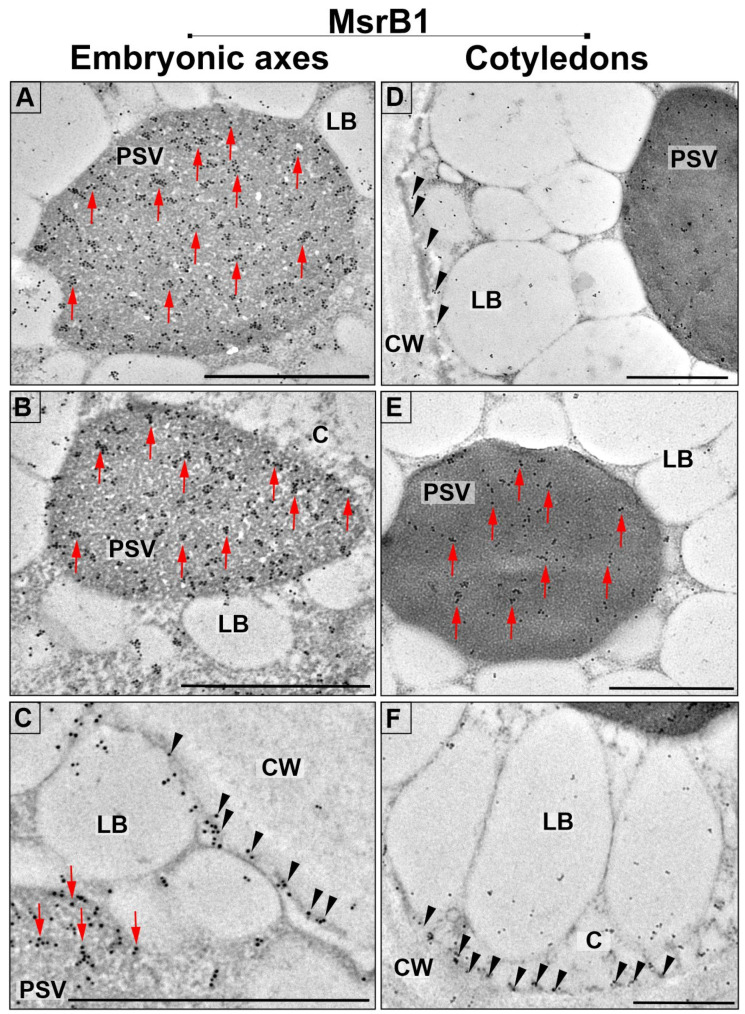
Subcellular localization of MsrB1 in the cells of embryonic axes (**A**–**C**) and cotyledons (**D**–**F**) of beech seeds: PSV—protein storage vacuoles, LB—lipid bodies, C—cytoplasm, and CW—cell wall; scale bars: 1 µm. Red arrows indicate the location of MsrB1 in PSVs, black arrowheads indicate the location of MsrB1 near the cell membrane.

Immunocytochemical labelling of MsrB proteins provided additional details regarding its subcellular localization. The MsrB1 proteins were detected in embryonic axes, primarily in protein storage vacuoles (PSVs) (Figure 3A–C, arrows). In addition, gold particles were also observed in the cytoplasm, especially in close proximity to the cell membrane (Figure 3C, arrowheads). A significantly lower level of gold particles was detected in cotyledons, in which the distribution of MsrB1 protein was similar to that observed in embryonic axes. The gold particles were localized primarily in PSVs (Figure 3D,E, arrows) and were located near the cell membrane (Figure 3F, arrowheads).

**Figure 4:**
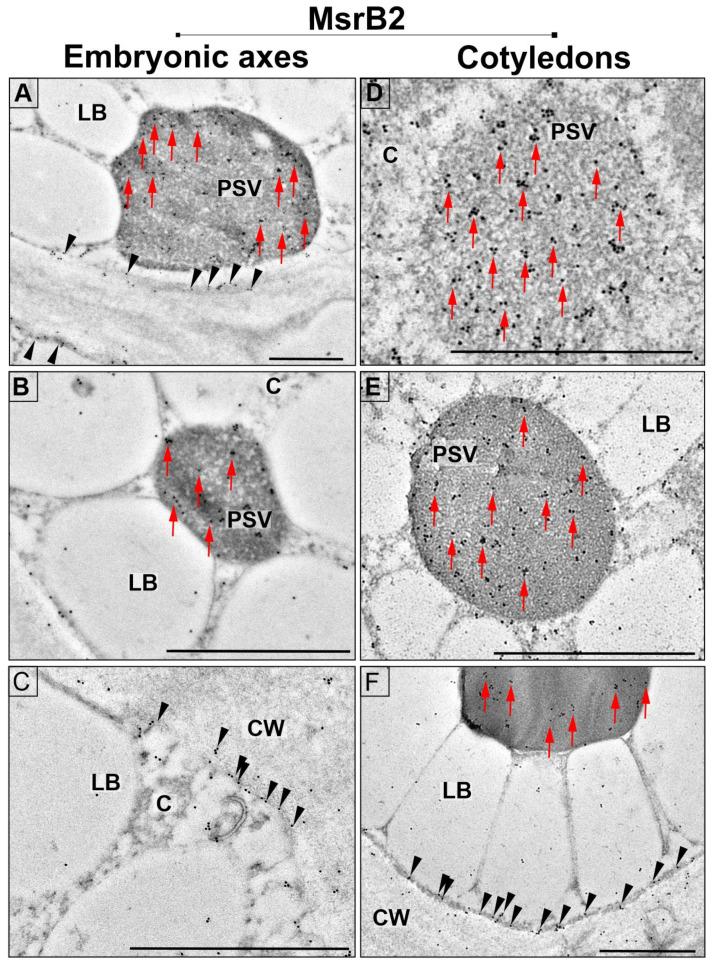
Subcellular localization of MsrB2 in the cells of embryonic axes (**A**–**C**) and cotyledons (**D**–**F**) of beech seeds: PSV—protein storage vacuoles, LB—lipid bodies, C—cytoplasm, and CW—cell wall; scale bars: 1 µm. Red arrows indicate the location of MsrB2 in PSVs, black arrowheads indicate the location of MsrB2 near the cell membrane.

The pattern of localization of MsrB2 in embryonic axes was similar to that of MsrB1; however, the number of particles was considerably smaller. The proteins were determined to be localized in electron-dense PSVs (Figure 4A,B, arrows) and scattered in the cytoplasm. The MsrB2 proteins were also observed to be localized along the plasma membrane (Figure 4C, arrowheads). In cotyledons, the pattern of signal distribution of MsrB2 was similar to that of MsrB1. The gold particles were primarily localized in PSVs (Figure 4D–F, arrows), and they were scattered in the cytoplasm (Figure 4D–F). In addition, the signal was also detected in the cytoplasm along the cell membrane (Figure 4F, arrowheads).

### 2.4. Bioinformatics Analyses

To address the question of how the two MsrB proteins can interact with the elements of PSVs, the cytoplasm, and the cellular membrane, a series of in silico predictions were made (Figures S1–S5).

#### 2.4.1. Protein–Membrane Interactions

Considering the low charge on the cell membranes, MsrB1 and MsrB2 were predicted to be located around the midplane in proximity of 70 Å and 68–86 Å, respectively, whereas the N-terminal parts of both proteins tended to stay in the aqueous phase (Figure S1A). The interaction of N-terminal parts with membranes containing 50% charged lipids was distinct, demonstrating that the MsrB1 protein can approach the phosphate groups of the lipid polar heads (20 Å) and that MsrB2 might even transverse the lipid bilayer (Figure S1B). The possibility of the formation of a transmembrane domain at the N-terminal part of the protein was predicted specifically for MsrB2 (Figure S2). Both MsrB proteins can undergo palmitoylation—a reversible covalent attachment of fatty acids, such as palmitic acid, to cysteine and, less frequently, to serine and threonine residues of proteins [36]. Using a high threshold, it was predicted that MsrB1 might be palmitoylated at Cys 116 and that MsrB2 can be palmitoylated at two residues, Cys 115 and Cys 116 (Figure S3). The peptides overlapping sites of palmitoylation were reconsidered in protein–membrane interactions, and regions ranging from 86 to 135 aa containing palmitoylation sites exactly in the middle were analysed again using the MCPep server; however, the distance did not shorten, probably because of low helical content, which ranged from 20 to 40%.

#### 2.4.2. Protein–Nucleic Acid and Protein–Protein Interactions

One DNA binding site, at the amino acid at position 35 in the protein sequence, and 10 protein binding sites or regions located at positions 30, 35–42, 52–60, 64–69, 101, 106, 147, 160, 171, 173, and 174 in the protein sequence were predicted for MsrB1, whereas one polynucleotide binding region, at the amino acid at position 38 in the protein sequence, and 13 protein binding sites or regions located at positions 1, 2, 28, 29, 42, 43, 52, 54–59, 62, 103, 153–155, 166, 177, 179, 182–184, and 202 in the protein sequence were predicted for MsrB2 (Figure S4). The interaction network predicted for the MsrB1 and MsrB2 proteins consisted of 10 and 11 functional partners, respectively (Figure S5). The protein partners were members of protein families related to methionine sulfoxide reductases, thioredoxins, glutaredoxins, and CAX-interacting protein 1 (CXIP1) (Table S1).

## 3. Discussion

The first plant gene encoding methionine sulfoxide reductase B (MsrB) was identified in Arabidopsis while researchers were searching for homologs to the human selenoprotein X proteins [37]. Since that study was published, numerous studies have shown that MsrB enzymes are important factors involved in the regulation of multiple processes during plant development [28,29] as well as in the response to biotic and abiotic stress [23,24,25,29]. Châtelain et al. [29] determined that the Msr repair system plays a decisive role in establishing and preserving seed longevity. The MsrB1 and MsrB2 proteins detected in stored beech seeds completely fit this description. In particular, the abundance of MsrB2 correlates well with the time of storage, suggesting that this Msr isoform is important in maintaining seed quality during long-term storage. Moreover, it has recently been reported that the MsrB2 protein is involved in seed desiccation tolerance [28]. Importantly, the loss of desiccation tolerance also results in the loss of seed viability [38]. These findings suggest that MsrB2 is involved in ensuring the extended longevity of beech seeds.

MsrB1 and MsrB2 proteins are thought to belong to chloroplast proteins [13]. This property was indicated by numerous studies that began in the 1980s, when it was confirmed that the chloroplastic fraction is characterized by 85% Msr activity [39]. Further research indicated that Arabidopsis has only 3 Msr chloroplastic isoforms, among 14 isoforms that were identified in this species, which are associated with these organelles. These isoforms were determined to be MsrB1, MsrB2, and MsrA4, but the latter exhibited considerably lower activity [23,40]. Interestingly, both beech seeds and *Acer* seeds [28] accumulate these proteins in cells lacking chloroplasts. Our microscopic analysis showed that MsrB1/B2 are present in several types of cells in both embryonic axes and cotyledons. The increased concentration of studied proteins was located in central vascular cylinder cells, primarily in outer-located areas related to developing conducting tissues, such as xylem cells, which may be associated with increased levels of ROS in these areas, as was described in aged beech seeds by Ratajczak et al. [41]. ROSs exhibit a two-sided nature, playing an important signalling role in many developmental processes in plants and, in contrast, being responsible for oxidative damage to cells and even cell death, especially when the balance between ROS production and scavenging is disturbed [8,42,43]. It is suggested that the abundance of MsrBs in ROS-enriched areas corresponding to developing conductive tissues may be related to the formation of xylem because increased levels of hydrogen peroxide and superoxide anion radical were reported during the xylogenesis process in the stem and roots of *Populus* [43]. Msrs in the central vascular cylinder may be associated with the protection of crucial enzymes, such as proteases, which are responsible for the degradation of cellular components and the formation of fully functional xylem vessels during programmed cell death accompanying xylogenesis. On the other hand, the signal distribution of Msr proteins may reflect sites where antioxidant functions of Msrs, including the prevention and repair of oxidative damage, are exhibited. The participation of Msrs in ROS scavenging was observed in seeds to limit the extent of damage resulting from highly oxidative conditions occurring during seed maturation and imbibition. In particular, plastidial isoforms of Msr were demonstrated to play crucial roles in the establishment and preservation of longevity in plant seeds [29]. This finding is in keeping with our results, which have shown that the abundance of Msrs decreases as seed storage time is increased. These results were especially pronounced in embryonic axes, in which a considerably higher concentration of ROS was confirmed compared to cotyledons [41]. ROS accumulation in aged embryonic axes may have an impact on the inability to form the primary root and to thus decrease germination capacity [44]. A decrease in Msr abundance accompanied by extended lipid peroxidation, imbalance in redox state, and DNA fragmentation may lead to cell death and, eventually, to a loss of viability in whole seeds [41,44].

Rouhier et al. [12] predicted the subcellular localization of A and B types of Msrs in different organs (roots, leaves, flower buds, and pollen) of *Arabidopsis thaliana*, providing examples of locations in the cytosol, plastids, endoplasmic reticulum, and secretory pathway. Since that study was published, no Msr isoform has been theoretically and/or experimentally proven to be located within PSVs. This study is the first to confirm that MsrB1 and MsrB2 accumulate in PSVs. Mature seeds contain densely packed storage protein deposits that are frequently observed to entirely fill the PSVs, which are the main structures responsible for accumulation and defence proteins [45]. Many regions of protein–protein interaction sites were predicted in the amino acid sequences of MsrB1 and MsrB2 (Figure S4), confirming the possibility of deposition of MsrBs within PSVs. In mature seeds, especially in dried seeds, chloroplasts undergo dedifferentiation or degeneration by several autophagic pathways [46]. Whole chloroplasts can be transported into vacuoles or cytoplasmic vesicles, known as Rubisco-containing bodies, which also can be carried into vacuoles [46,47]. It is possible that chloroplast stroma degradation elements, including plastidial MsrB1 and MsrB2, were transported into vacuoles and further conveyed into PSVs. PSVs break down during germination; therefore, there is a possibility that accumulated MsrB1/B2 are a pool of proteins that participate in the regulation of seed germination. Proper storage reserve mobilization is essential for germination initialization and normal seedling establishment [48]. Beech seed germination manifested by embryonic axis elongation begins at approximately the 12th week of cold stratification [5]. Importantly, in beech seeds subjected to germination, protein storage vacuoles have been reported to diminish or disappear after the first week of stratification and were mostly utilized at the final stage of stratification [49]. In this context, MsrB1 and MsrB2 are suggested to be involved in proper storage material utilization in beech seeds prior to germination. Moreover, in germinating beech seeds, considerable accumulation of ROS was reported, particularly in elongating embryonic axes [50]. Overproduction of ROS may cause chemical damage to nucleic acids, lipids, and proteins, especially those with sulphur-containing amino acids [51,52]. This effect may indicate the need for the presence of active protective mechanisms in response to the increase in ROS concentration. Less abundant MsrB proteins in long-term stored beech seeds probably contribute less to protection. Water restriction in the dry state limits molecular mobility [53]; consequently, Msr localization would not change during storage but Msr abundance would clearly decline.

Except for the accumulation of MsrB1 and MsrB2 in PSVs, these proteins were reported to be abundant in the cytoplasm, primarily in close vicinity to the plasma membrane. Interestingly, to date, no articles describing the interaction of plant Msr with membrane components have been published. It has not been determined whether membrane lipids or membrane proteins are the primary targets of MsrBs. In silico predictions confirmed that both MsrB proteins can interact with membrane phospholipids (Figures S1 and S2). The N-terminal part of MsrB1 in particular can interact with the phosphate groups of the lipid polar heads, whereas the N-terminal part of MsrB2 was predicted to form a small transmembrane region (Figure S2) and to transverse the charged membrane bilayer (Figure S1). Both sides of the membrane surface are negatively charged [54], thereby increasing the accuracy of our predictions. Charged lipids can modulate proteins using two primary mechanisms: specific lipid–protein interactions or membrane-mediated interactions [55]. In this context, the interaction of MsrB1 and, predominantly, MsrB2 with phospholipids is highly possible and might explain the protein location detected near the cellular membrane. Additionally, palmitoylation, which is a highly probable posttranslational modification of MsrB1 and MsrB2 (Figure S3), by enhancing the hydrophobicity of proteins, by modulating the association of proteins with the membrane, and by modulating protein–protein interactions, may influence subcellular trafficking of MsrB proteins between membrane compartments after seed hydration. Palmitoylation and depalmitoylation enable the shuttling of proteins between different organelles, cellular regions, and membranous compartments [56], further supporting our hypothesis. Importantly, putative palmitoylation at Cys-115 and/or Cys-116 would not inhibit either coordination of the zinc atom linked to Cys positioned at 187 in MsrB2 and Cys-186 in MsrB1 or the catalytic function of MsrB2 because the resolving Cys is positioned at 134 in MsrB2 [19]. Interestingly, the cytosolic form of mouse MsrA undergoes myristoylation, another example of protein lipidation that is associated with the targeting of MsrA to two distinct cellular compartments [57].

Cell membranes are regarded as the main site of seed desiccation injury [58] because disorganization of membranes results in electrolyte leakage, which is an important factor that influences the storability of beech seeds [59]. In this context, MsrBs located in close proximity to the cellular membrane could be involved in the neutralization of oxidative stress accompanying tissue drying, thereby enabling membrane stabilization in the dry state because lipid peroxidation is strongly related to senescence of beech seeds [10]. In particular, MsrB2 might be involved in the protection of dry beech seeds because the pepper *MsrB2* gene has been proven to confer drought tolerance [24].

Among Arabidopsis Msrs, 9 isoforms (A1–A3 and B4–B9) were predicted to be localized in the cytosol [12]. The two proteins, MSRB7 and MSRB8, were experimentally localized in the cytosol, where they were assumed to play a role in defence against oxidative stress [14]. MsrB1 and MsrB2 were detected in the cytoplasm of cells of beech seeds, probably because they were synthesized there, but the lack of active chloroplasts [25,60] or possibly the masking of signal peptides determining their targeting to chloroplasts [34] enabled them to remain in the cytoplasm, where they can exhibit their antioxidant functions. Additionally, MsrB1 and MsrB2 were reported to surround spherical structures (Figure 2K), which might be an example of the interaction between their signal peptides with specific receptors located at the surface of proplastids that will develop into chloroplasts in the future. The presence of *Capsicum annuum* MsrB2 protein (CaMsrB2) in the cytoplasm was also confirmed in transformed protoplasts of *Nicotiana benthamiana* [25]. In addition to the cytoplasm, CaMsrB2 protein also localized in the nucleus. This localization was not confirmed experimentally in seeds; however, in silico predictions clearly indicated the presence of one DNA binding site and one polynucleotide binding region in the amino acid sequences of MsrB1 and MsrB2, respectively [41].

The fact that *Glycine soja* methionine sulfoxide reductase B5a can interact with a kinase, thereby activating the ROS signalling pathway, motivated us to find possible functional partners of MsrB1 and MsrB2 [61] (Figure S5 and Table S1). The amino acid composition determines the possibility of the formation of additional bonds that can be used to interact with other molecules; thus, it was possible to identify functional partners of MsrB1 and MsrB2 (Figure S5). MsrB2 and other 2-Cys Msrs are regenerated through a thioredoxin (Trx)-dependent mechanism, whereas MsrB1 regeneration employs glutaredoxin (Grx)- or GSH/Grx-dependent mechanisms [62,63]. Glutaredoxins were predicted as specific partners uniquely associated with MsrB1 (Figure S5). Interestingly, CXIP1, the functional partner of MsrB1, may only reduce GSH-thiol disulphides but not protein disulphides. Glutathione was suggested as an important ligand involved in regeneration of MsrB1 [19]. It is possible that an MsrB1-GSH-CXIP1 interaction is required for efficient MsrB1 regeneration. Interestingly, several other isoforms of Msrs were predicted to be functional partners of both MsrB1 and MsrB2 (Table S1), thereby possibly explaining the similar tissue and subcellular localization of MsrB1 and MsrB2 reported in this study (Figure 2, Figure 3 and Figure 4).

Based on controlled deterioration experiments on *A. thaliana* and *M. truncatula* seeds, with both species producing the orthodox category (desiccation tolerant) seeds, Msr enzymatic capacity appeared to be strongly linked to seed longevity. In this study, MsrB1 and MsrB2, both considered to be plastidial proteins, were observed in beech seeds and their abundance significantly decreased during long-term storage. The tissue and subcellular localization of Msrs were examined to determine their possible role in seed viability. The newly discovered location of MsrB1 and MsrB2 within PSVs might be particularly associated with beech seed longevity via the protection of storage material utilization machinery during germination. These findings, combined with those of our previous reports, suggest that MsrB2 is a multifunctional protein that plays a role in redox regulation during seed development, seed desiccation, and long-term storage with implications for seed germination.

## 4. Materials and Methods

### 4.1. Plant Material

Seeds of the European beech (*Fagus sylvatica* L.) stored for 2, 10, 13, 16, and 19 years displaying 92, 82, 81, 67, 30% germination capacity, respectively, were analysed. Dry seeds (below 10% of water content) were stored in plastic containers at −10 °C [6]. The seed coats were removed, and the embryonic axes were separated from cotyledons before performing experiments. For all microscopic analyses, seeds were hydrated prior to fixation.

### 4.2. Protein Extraction and Western Blotting

For each experimental variant, twenty embryonic axes and five cotyledons were ground to a powder in liquid nitrogen in a chilled mortar and pestle. The dry powder was incubated for one hour with buffer containing 25 mM Tris-HCl, 5% glycerol, 3 mM β-mercaptoethanol, and 2% polyvinylpolypyrrolidone at 4 °C, with shaking every 15 min and centrifugation for 20 min at 20,000× *g* at 4 °C. The protein concentration was measured using Bradford assay [64]. Proteins were separated by SDS-PAGE on 12% polyacrylamide gels, with an equal amount of protein (20 μg) in each lane (Figure S6). Western blot analysis was performed according to the method described by Wojciechowska et al. [28]. Primary antibodies against MsrB1 and MsrB2 [20] were diluted 1:1000 in 5% skimmed milk. Antibodies specific to Arabidopsis MsrB1 and MsrB2 proteins were recognized in *Acer* seeds with single bands of 17 kDa and 15 kDa, respectively [28] similar to *Arabidopsis* [20]. Secondary antibodies conjugated with horseradish peroxidase (HRP, Agrisera, Sweden catalogue number AS09 602) were diluted 1:10,000 in 5% skimmed milk. Wb images were analysed densitometrically in triplicate using the UviBand (UviTec, Cambridge, UK) program of the Fire Reader Gel Documentation System. The band density was calculated based on the volume (V) of the band as the sum of all 3D intensities (I) coded on a scale of 256 grey levels. The data are presented in relative units obtained from V = Σn_i_I and the number of pixels inside the area of the band.

### 4.3. Anatomical Studies

For histological analyses, at least ten embryonic axes and cotyledons were fixed and embedded in Technovit resin (Heraeus Kulzer, Wehrheim, Germany) according to the protocol described by Wojciechowska et al. [65]. Cross sections were cut with a Leica RM2265 Fully Automated Rotary microtome (Leica-Reichert, Bensheim, Germany) at a thickness of 12 μm. The embryonic axes were cut along their entire length, while those fragments in which tissues were differentiated (usually 1.5 mm from the tip) are shown on the figures. The cross sections were stained with 0.1% (*m*/*v*) toluidine blue (pH 4.4) examined under a light microscope (LM) using ZEN microscope software (Carl Zeiss, Jena, Germany).

### 4.4. Immunolocalization of MsrB

#### 4.4.1. Immunofluorescence

Cotyledons fragmented to 5-mm × 5-mm pieces and the whole embryonic axes were fixed in a mixture of 2% glutaraldehyde (Polysciences, Warrington, FL, USA) and 2% (*v*/*v*) formaldehyde (Polysciences, Warrington, FL, USA) for 12 h at 4 °C. Then, the material was rinsed three times in 1× phosphate buffer saline (PBS) (Sigma, St Louis, MO, USA) buffer and sectioned (30 µm) using a Leica VT 1200S vibratome (Leica Biosystems, Nussloch, Germany). Primary rabbit antibodies against MsrB1 and MsrB2 at a dilution of 1:100 were used for localization of MsrB1 and MsrB2 proteins. The TSA technique (Thermo Fisher Scientific Inc., Waltham, MA, USA) was applied because of its much higher sensitivity compared to the standard protocol of the immunolocalization method. All steps of the immunofluorescent reaction were performed as described by Wojciechowska et al. [66]. The localization results were analysed and documented with a Leica TCS SP5 confocal microscope (Leica Biosystems, Nussloch, Germany) using an argon laser emitting light at wavelengths 488 for Alexa Fluor 488. For each organ, the analysis was performed in six biological replications. Negative control reactions without primary antibodies were performed in triplicate (Figure S7).

#### 4.4.2. Immunocytochemistry

Fragments of cotyledons (2-mm × 2-mm) and embryonic axes were fixed in 0.5% glutaraldehyde (Polysciences, Warrington, FL, USA) and 4% formaldehyde (Polysciences, Warrington, FL, USA) in 0.1 M sodium cacodylate buffer (pH 7.2) (Polysciences, Warrington, FL, USA) for 12 h at 4 °C. Fixed samples were dehydrated in graduated ethanol concentrations and embedded in LR White resin (Sigma, St Louis, MO, USA). Ultrathin sections (60 nm) were cut on a Leica EM UC7 (Leica, Nussloch, Germany) ultramicrotome using a diamond knife, sections were collected on formvar-coated nickel grids, and the material was blocked in 1% acetylated BSA (acBSA) in PBS for 15 min at room temperature. After blocking, sections were incubated with a primary rabbit antibody against methionine sulfoxide reductases, both isoforms MsrB1 and MsrB2. The primary antibodies were diluted 1:20 in 0.05% acBSA. After washing in PBS, the sections were incubated with 10 nm gold-labelled goat anti-rabbit secondary antibody (Sigma, St. Louis, MO, USA) diluted 1:20 in 0.05% acBSA in PBS at 37 °C for 2 h. Then, sections were stained with 2% uranyl acetate for 15 min and examined with a Hitachi HT7700 transmission electron microscope (Hitachi, Tokyo, Japan) operating at an accelerating voltage of 80 kV. For cytological studies, ten embryonic axes and cotyledons were embedded, and at least three copper grids for each organ were examined under an electron microscope. In the control reactions, incubations with the primary antibodies were omitted (Figure S8).

### 4.5. Bioinformatic Analyses

Two methionine sulfoxide reductase proteins originating from *Arabidopsis thaliana,* MsrB1 (accession AEE32980.1) and MsrB2 (accession OAO98568.1), were employed as queries. The MCPep server (http://bental.tau.ac.il/MCPep/) was used for Monte Carlo simulations of peptide–membrane interactions [67]. The possibility of amino acid interactions with lipid membranes was calculated as the distance from the membrane midplane and presented in Å units. The Phobius server was used for prediction of transmembrane topology and signal peptides from the amino acid sequence of a protein [68]. The probability of MsrB1 and MsrB2 palmitoylation was assessed using CSS-Palm Online Service (http://csspalm.biocuckoo.org/online.php) [36]. Profisis [69], a part of the Predict Protein Server (https://www.predictprotein.org/), a machine learning-based method that identifies interacting residues from sequences alone using transient protein–protein interfaces from complexes of experimentally known 3D structures, was used to predict potential protein–protein interactions. The protein interaction network was constructed using the STRING (string-db.org) database [70].

### 4.6. Statistical Analyses

All experiments were performed with three independent biological replicates. Statistically significant differences were indicated with different letters (ANOVA and Tukey’s test at *p* > 0.05).

## Data Availability

The data that supports the findings of this study are available from the corresponding author upon reasonable request.

## Supplementary Materials

Supplementary Materials can be found at https://www.mdpi.com/1422-0067/22/1/402/s1. Figure S1: Location of the MsrB1 and MsrB2 near the membrane, Figure S2: Prediction of transmembrane topology, Figure S3: Prediction of sites of palmitoylation, Figure S4: Prediction of protein–nucleotide and protein–protein interaction sites, Figure S5: Protein–protein interaction networks, Figure S6: Representative gels, Figure S7: Negative controls of immunofluorescent reactions, Figure S8: Negative controls of immunogold labelling, Table S1: Functional protein partners of MsrB1 and MsrB2.

## Abbreviations

Cys	Cysteine
Grx	Glutaredoxin
HRP	Horseradish peroxidase
LBs	Lipid bodies
Met	Methionine
MetO	Methionine sulfoxide
Msr	Methionine sulfoxide reductase
PBS	Phosphate-buffered saline
PSV	Protein storage vacuoles
ROS	Reactive Oxygen Species
TSA	Tyramine Signal Amplification
Trx	Thioredoxin
WC	Water content
