# Supplementary material for: Localization and Dynamics of the Methionine Sulfoxide Reductases MsrB1 and MsrB2 in Beech Seeds
Source: Int J Mol Sci. 2021 Jan 2;22(1):402. doi: 10.3390/ijms22010402 (PMC7795007; doi:10.3390/ijms22010402)
Supplement: Supplementary file 1 [file ijms-22-00402-s001.pdf]

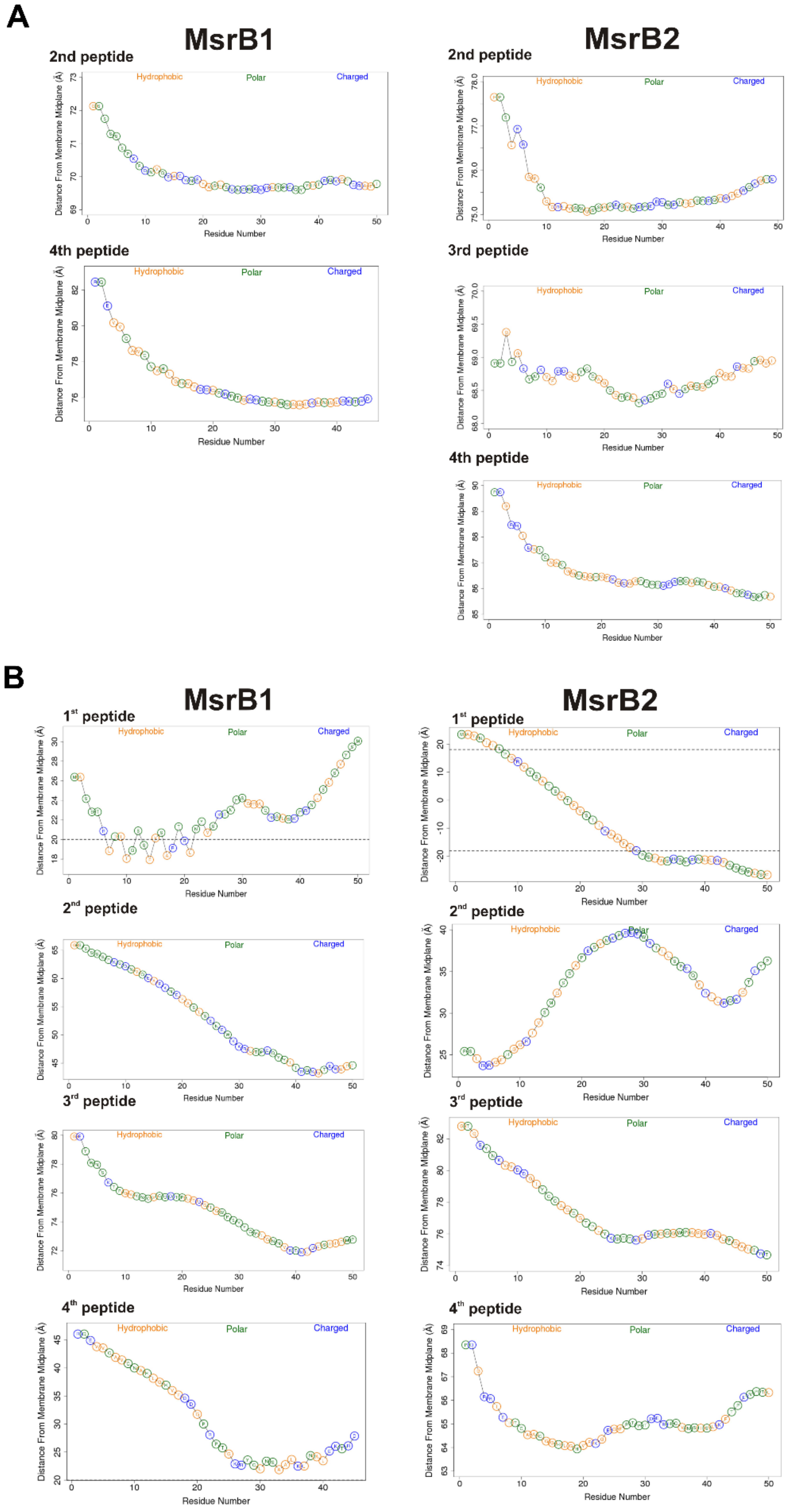

Figure S1 The average location of the amino acids of MsrB1 and MsrB2 near the membrane. The membrane included A) 30%-charged lipids and B) 50%-charged lipids. The location of the phosphate groups of the lipid polar heads reaches 20 Å. The hydrophobic residues (G, A, V, L, I, F) are in orange, polar residues (M, C, T, S, W, Y, H, Q, N) in green and charged amino acids (R, K, E) in blue.

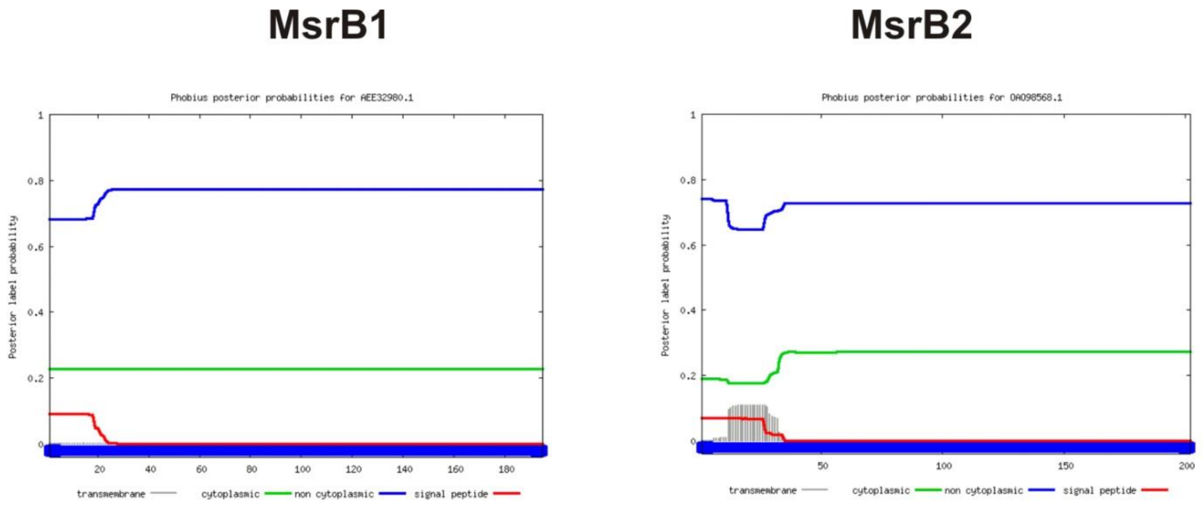

Figure S2 Prediction of transmembrane topology and signal peptides from the amino acid sequence of MsrB1 and MsrB2 protein based on Phobius server (Käll et al. 2007).

AEE32980.1 methionine sulfoxide reductase B 1 [Arabidopsis thaliana]

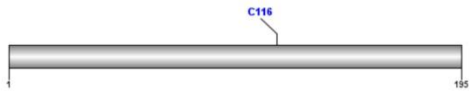

ID	Position	Peptide	Score	Cutoff
AEE32980.1 methionine sulfoxide reductase B 1 [Arabidopsis thaliana]	116	PGVYNCVCCDTPLFD	5.7	3.419

OAO98568.1 MSRB2 [Arabidopsis thaliana]

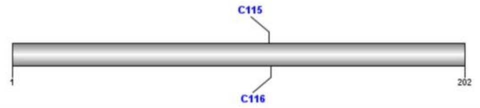

ID	Position	Peptide	Score	Cutoff
OAO98568.1 MSRB2 [Arabidopsis thaliana]	115	VFDDGIYCCAGCGTP	6.878	3.419
OAO98568.1 MSRB2 [Arabidopsis thaliana]	116	FDDGIYCCAGCGTPL	6.081	4.222

Figure S3 Prediction of sites of palmitoylation, a post-translational modification based on reversible covalent attachment of fatty acids to cysteine in the amino acid sequence of MsrB1 and MsrB2 using CSS-Palm Online Service (Ren et al. 2008).

.

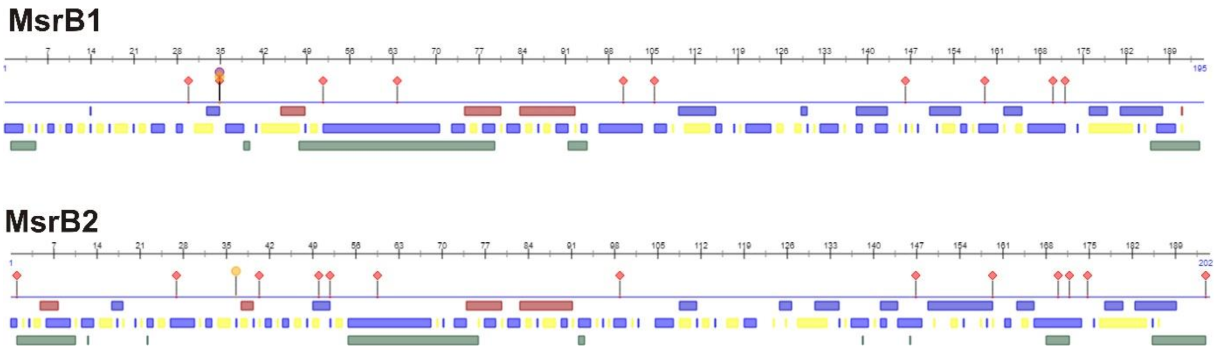

Figure S4. Prediction of DNA and polynucleotide binding sites as well as protein-protein interaction sites in the amino acid sequence of MsrB1 and MsrB2 using Profisis service (Ofran and Rost 2007). Protein binding sites are pointed with red diamonds and nucleotide binding sites are pointed with yellow circle and described below as a single residues or tracks of residues in the amino acid sequence. Line consisting of red and blue rectangles corresponds to secondary structure of protein and represent helix and strand, respectively. Line consisting of yellow and blue rectangles corresponds to buried and exposed, respectively, amino acids. Green rectangles correspond to disordered regions.

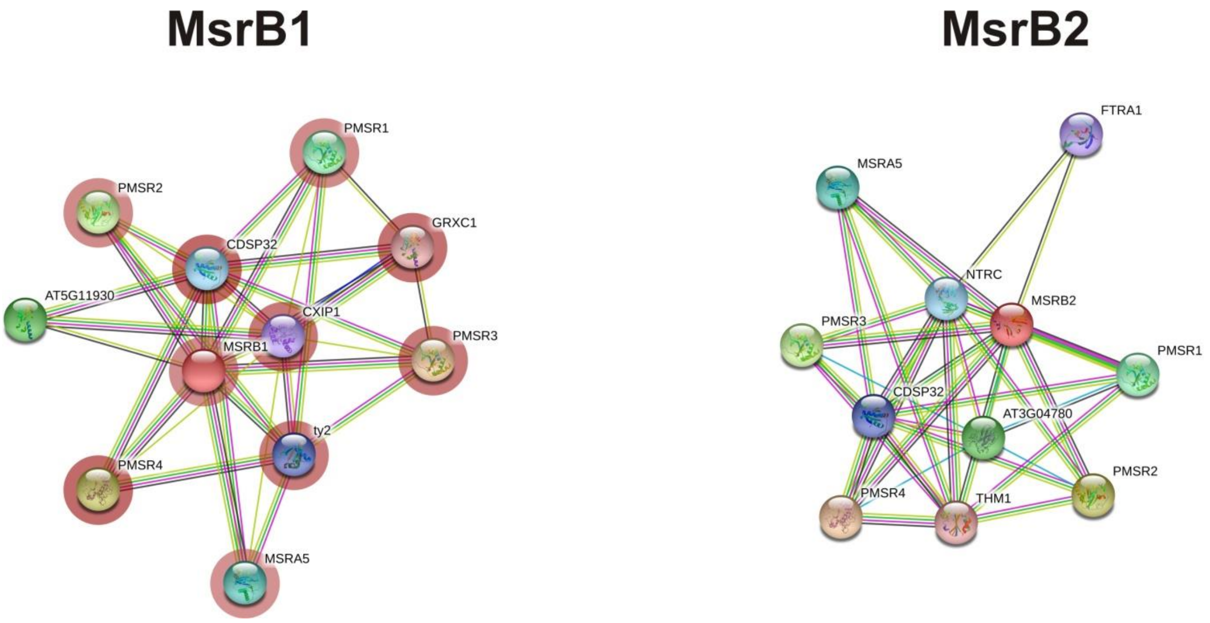

Figure S5. Protein-Protein interaction networks performed for MsrB1 and MsrB2 proteins and STRING functional enrichment analysis. Red line - indicates the presence of fusion evidence. Green line - neighborhood evidence. Blue line - cooccurrence evidence. Purple line - experimental evidence. Yellow line - text mining evidence. Light blue line - database evidence. Black line - coexpression evidence. In confidence mode the thickness of the line indicate the degree of confidence prediction of the interaction.

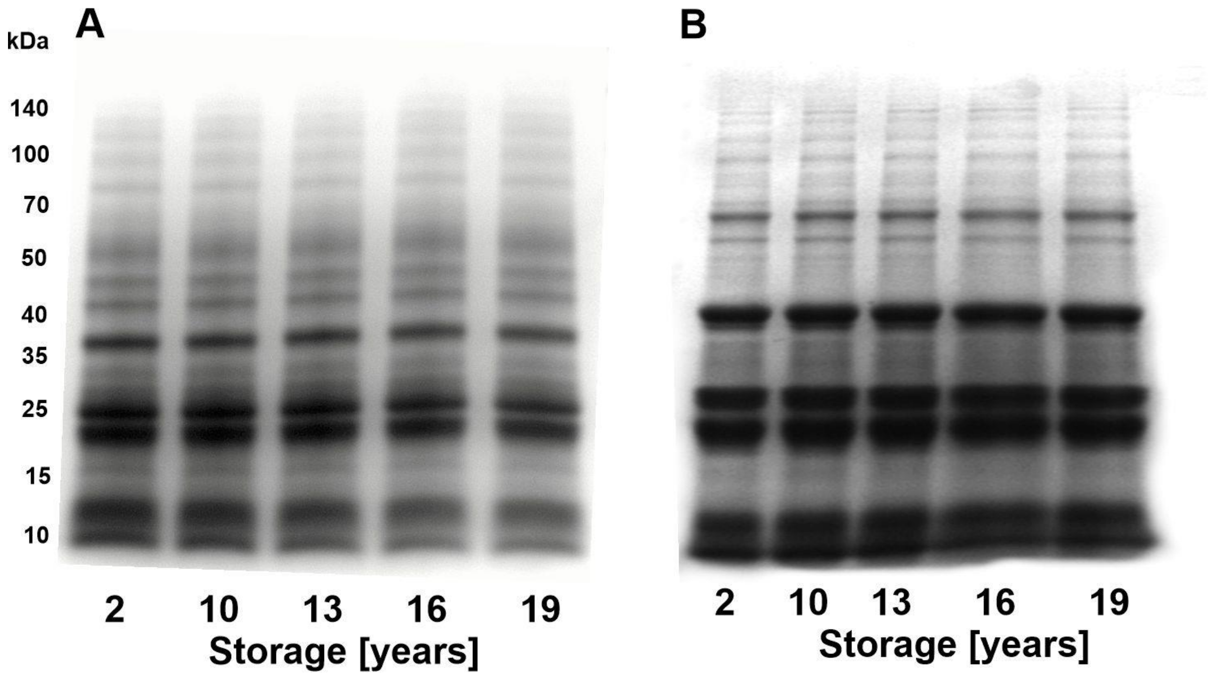

Figure S6. SDS-PAGE representative gels of protein extracts (20 μg) isolated form embryonic axes (A) and cotyledons (B) of beech seeds further used for Western blot analyses. Analyses concerned proteins isolated from seeds stored for 2, 10, 13, 16 and 19 years. The SpectraTM Multicolor Broad Range Protein Ladder marker (Thermo Scientific) was used to calculate the molecular weight. Gels were documented with using G:BOX Chemi XR5 instrument (Syngene, Cambridge, UK) and Coomassie Blue filter settings.

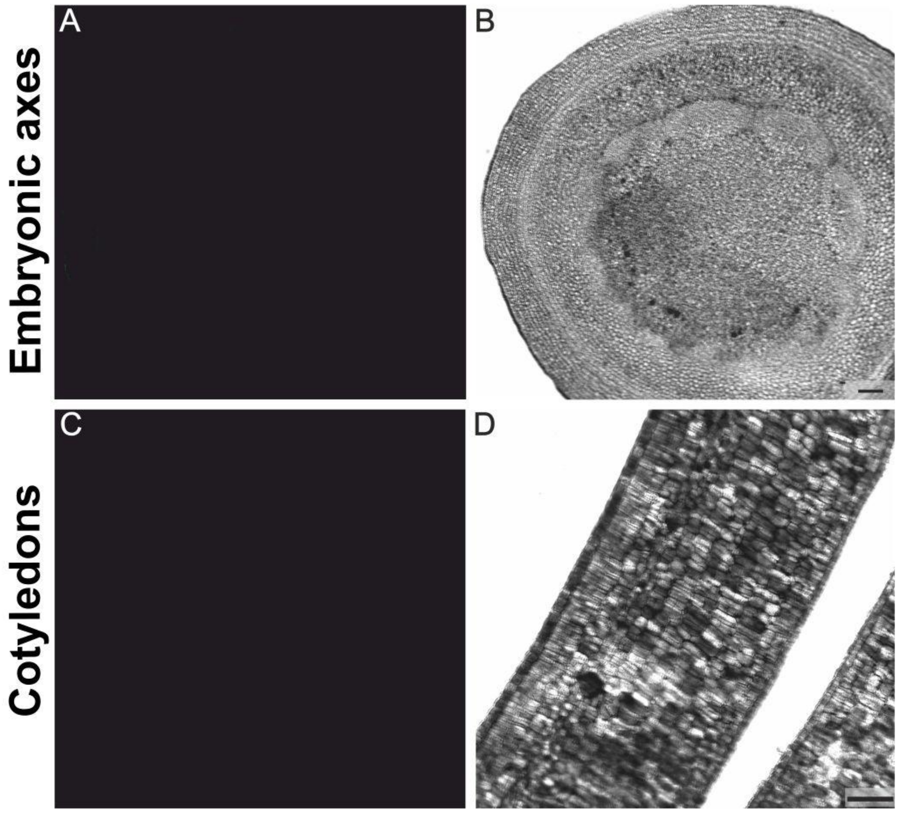

Figure S7. Negative controls of immunofluorescent reactions in embryonic axes (A) and cotyledons (C). Scale Bars=100 μm.

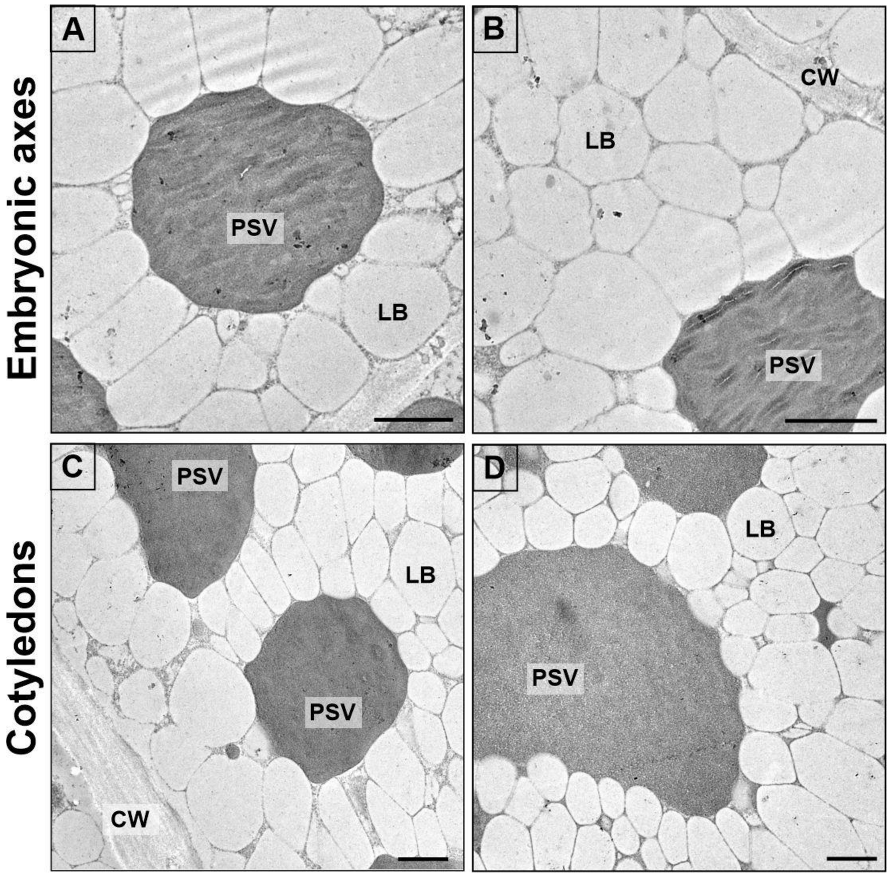

Figure S8. Negative controls of immunogold labelling in embryonic axes (A,B) and cotyledons (C,D). Scale Bars=1 μm.

Table S1

Functional protein partners of MsrB1 and MsrB2 predicted by STRING server and sortedin groups of thioredoxins, glutaredoxins and others.

MsrB1 Functional Partners with scores			
**Methionine sulfoxide reductases**	**Thioredoxins**	**Glutaredoxins**	**other**
PMSR3 0.951<br>PMSR4 0.930<br>PMSR2 0.920<br>PMSR1 0.872<br>MSRA5 0.872	CDSP32 0.860<br>ty2 0.816	AT5G11930 0.875<br>GRXC1 0.705	CXIP1 0.714
**MsrB2 Functional Partners with scores**			
**Methionine sulfoxide reductases**	**Thioredoxins**		
PMSR4 0.933<br>PMSR2 0.922<br>PMSR3 0.920<br>PMSR1 0.872<br>MSRA5 0.872	NTRC 0.764<br>CDSP32 0.739<br>FTRA1 0.709<br>THM1 0.67<br>CDSP32 0.739<br>AT3G04780 0.900		
